# Ensemble-based prediction of RNA secondary structures

**DOI:** 10.1186/1471-2105-14-139

**Published:** 2013-04-24

**Authors:** Nima Aghaeepour, Holger H Hoos

**Affiliations:** 1 Terry Fox Laboratory, British Columbia Cancer Agency, Vancouver, BC, V5Z 1L3, Canada; 2 Department of Computer Science, University of British Columbia, Vancouver, BC, V6T 1Z4, Canada

## Abstract

**Background:**

Accurate structure prediction methods play an important role for the understanding of RNA function. Energy-based, pseudoknot-free secondary structure prediction is one of the most widely used and versatile approaches, and improved methods for this task have received much attention over the past five years. Despite the impressive progress that as been achieved in this area, existing evaluations of the prediction accuracy achieved by various algorithms do not provide a comprehensive, statistically sound assessment. Furthermore, while there is increasing evidence that no prediction algorithm consistently outperforms all others, no work has been done to exploit the complementary strengths of multiple approaches.

**Results:**

In this work, we present two contributions to the area of RNA secondary structure prediction. Firstly, we use state-of-the-art, resampling-based statistical methods together with a previously published and increasingly widely used dataset of high-quality RNA structures to conduct a comprehensive evaluation of existing RNA secondary structure prediction procedures. The results from this evaluation clarify the performance relationship between ten well-known existing energy-based pseudoknot-free RNA secondary structure prediction methods and clearly demonstrate the progress that has been achieved in recent years. Secondly, we introduce AveRNA, a generic and powerful method for combining a set of existing secondary structure prediction procedures into an ensemble-based method that achieves significantly higher prediction accuracies than obtained from any of its component procedures.

**Conclusions:**

Our new, ensemble-based method, AveRNA, improves the state of the art for energy-based, pseudoknot-free RNA secondary structure prediction by exploiting the complementary strengths of multiple existing prediction procedures, as demonstrated using a state-of-the-art statistical resampling approach. In addition, AveRNA allows an intuitive and effective control of the trade-off between false negative and false positive base pair predictions. Finally, AveRNA can make use of arbitrary sets of secondary structure prediction procedures and can therefore be used to leverage improvements in prediction accuracy offered by algorithms and energy models developed in the future. Our data, MATLAB software and a web-based version of AveRNA are publicly available at http://www.cs.ubc.ca/labs/beta/Software/AveRNA.

## Background

RNAs are amongst the most versatile and oldest biomolecules; they play crucial roles in many biological processes. As in the case of proteins, the function of many types of RNAs critically depends on the three-dimensional structure of the molecules. However, the 3D structure of RNAs is determined to a larger degree by their secondary structure, which arises from base-pairing interactions within an RNA strand and stacking of the resulting base pairs.

Since the direct determination of 3D structures is difficult and costly, computational structure prediction methods, and in particular, secondary structure prediction methods, are widely used. A prominent and versatile approach for predicting RNA secondary structures is based on thermodynamic models, such as the Turner model [1], and uses dynamic programming algorithms (such as the Zuker & Stiegler algorithm [2]), to find a structure with minimum free energy (MFE) for a specific RNA sequence. Over the last five years, considerable improvements in the predictions obtained by such algorithms have been achieved.

It is important to note that, while it might seem natural to use experiments to determine the parameters of a thermodynamic model and machine learning and optimisation to determine those of a stochastic model, because of the equivalence between the free energy and probability of RNA structures, in principle, both approaches can be used in either setting. Indeed, the largest improvement in prediction accuracy has resulted from the use of sophisticated methods for estimating the thermodynamic parameters of a given energy model (in particular, the Turner model), based on a set of reliable RNA secondary structures [3-5]. Particularly good results have been achieved for methods in which parameter estimation additionally takes into account thermodynamic data from optical melting experiments, such as CG, LAM-CG and BL, [4,5] and expand the standard energy model with probabilistic relationships between structural features (*e.g.*, hairpin loops of different lengths), such as BL-FR [5]. Improved prediction accuracy has also been reported for an approach that determines structures with maximum expected accuracy (MEA) rather than minimum free energy, based on base pairing probabilities obtained from a partition-function calculation [3,6,7]. *CONTRAfold* implements a conditional log-linear model (which generalizes upon stochastic context-free grammars) for structure prediction. MaxExpect starts from base-pair probabilities calculated by partition functions [8] and uses dynamic programming (similar to *CONTRAfold*) to predict the MEA structure [6]. And finally, CentroidFold uses a similar strategy except that it uses a weighted some of true positives and true negatives as the objective function [7].

While the overall improvement in accuracy achieved over the baseline provided by the Zuker & Stiegler algorithm using the Turner model is clearly significant, there is less certainty about the more modest performance relationships between some of the more recent methods. For example, Lu *et al.* reported a difference of only 0.3% in average sensitivity between their MaxExpect procedure and *CONTRAfold 2.0*[3]. Similarly, Andronescu *et al.* found a 0.5% difference in average F-measure between *CONTRAfold 2.0* and their CG ^∗^ procedure [5]. Whether such small performance differences can be considered significant is an open question; in fact, a cross-validation experiment for the BL and LAM-CG parameter estimation methods suggests that 3% differences in accuracy may be statistically significant, but the evidence is far from conclusive [5]. This suggests that there is a need for methods that make it possible to assess the statistical significance of differences in prediction accuracy observed between RNA secondary structure prediction methods. In this work we present such methods, based on two well-established resampling techniques from statistics, bootstrapped confidence intervals and permutation tests. Using these methods and a well-studied, large set of trusted RNA secondary structures, we assess progress and the state of the art in energy-based, pseudoknot-free RNA secondary structure prediction.

Also, it has been demonstrated that the accuracies of predictions based on their BL ^∗^, CG ^∗^ and Turner99 parameter sets (see their Supplementary Results C) are not consistent across large and diverse sets of RNAs, and that differences in accuracy for many individual RNAs often deviate markedly from the average accuracy values measured across the entire set [5]. This suggests that by combining the predictions obtained from different procedures, better results can be achieved than by using any one of the given procedures in isolation. This general idea has been previously applied to a wide range of problems in computing science (where it underlies the fundamental approaches of boosting and bagging [9]). More recently, it has been used successfully for solving various problems from computational biology, including gene prediction [10], clustering protein-protein interaction networks [11], as well as analysis of data from microarrays [12] and flow cytometry [13].

Here, we introduce a generic RNA secondary structure prediction procedure that, given an RNA sequence, uses an ensemble of existing prediction procedures to obtain a set of structure predictions, which are then combined on a per-base-pair-basis to produce a combined prediction. Empirical analysis demonstrate that this ensemble-based prediction procedure, which we dub *AveRNA*, outperforms the previous state-of-the-art secondary structure prediction procedures on a broad range of RNAs. On the S-STRAND2 dataset [14], *AveRNA* obtained an average F-measure of 71.6%, compared to the previous best value of 70.3% achieved by BL-FR ^∗^[5]. *AveRNA* can easily be extended with new prediction procedures; furthermore, it provides an intuitive way of controlling the trade-off between false positive and false negative predictions. This is useful in situations where high sensitivity or high PPV may be required and allows *AveRNA* to achieve a sensitivity of over 75% and a PPV of over 83% on S-STRAND2.

## Methods

In this section, we first describe the data set and prediction accuracy measures used in our work. Next, we introduce the statistical methodology for the empirical assessment of RNA secondary structure prediction algorithms we developed in this work. This is followed by a brief summary of the set of procedures for MFE-based pseudoknot-free RNA secondary structure prediction we used in this work. Finally, we present *AveRNA*, our novel RNA secondary structure prediction approach, which combines predictions obtained from a diverse given set of procedures by means of weighted per-base-pair voting.

### Data sets

In this work, we used the S-STRAND2 dataset [14], which consists of 2518 pseudoknot-free secondary structures from a wide range of RNA classes, including ribosomal RNAs, transfer RNAs, transfer messenger RNAs, ribonuclease P RNAs, SRP RNAs, hammerhead ribozymes and group 1/2 introns [15-20]. This large and diverse set is comprised of highly accurate structures and has been used for the evaluation of secondary structure prediction accuracy in the literature [5]. For the parts of our work involving the optimization of prediction accuracy, in order to avoid overfitting, we used a subset of the S-STRAND2 dataset obtained by sampling 500 structures uniformly at random as the basis for the optimization process, and the full S-STRAND2 dataset for assessing the resulting, optimized prediction procedures.

### Existing secondary structure prediction methods

We used 10 secondary prediction procedures known from the literature. The SimFold- *V*2.0 procedure [21], which is based on Zuker and Stiegler’s classic dynamic programming algorithm, was used to predict secondary structures using six different sets of free energy parameters: Turner99 [1]; NOM-CG [4]; DIM-CG [22]; CG ^∗^, BL ^∗^ and BL-FR ^∗^[5]. Furthermore, we used *CONTRAfold*- *v*1.1, *CONTRAfold*- *v*2.0 [3], MaxExpect- *v*5.1 [6] and CentroidFold- *v*0.0.9 [7]. The two versions of *CONTRAfold* as well as CentroidFold are based on probabilistic methods that do not make use of physically plausible thermodynamic models of RNA secondary structure, while the seven other procedures are all based on (variations of) the widely used free energy model by the Turner group [1].

While we originally also considered taveRNA [23] and SARNA-Predict [24], it turned out to be infeasible to run these procedures on the the longer sequences from the S-STRAND2 dataset (due to runtime and memory requirements).

### Accuracy measures

Consistent with existing work on RNA secondary structure prediction, we assessed the prediction accuracy achieved by a given RNA secondary structure prediction procedure based on a given set of references structures, using sensitivity, positive predictive value (PPV) and the F-measure. We define a correctly predicted base-pair to be a predicted base-pair, exactly identical to one of the base-pairs in the reference structure. For a single RNA (sequence, structure) pair, sensitivity is the ratio of number of correctly predicted base-pairs to the number of base-pairs in the reference structure:

(1) $$\text{Sensitivity}=\frac{\#\text{Correctly Predicted Base-Pairs}}{\#\text{Base-Pairs in the Reference Structure}};$$

PPV is the ratio of number of correctly predicted base-pairs to the number of base-pairs in the predicted structure:

(2) $$\text{PPV}=\frac{\#\text{Correctly Predicted Base-Pairs}}{\#\text{Base-Pairs in the Predicted Structure}};$$

and the F-measure is defined as the harmonic mean of sensitivity and PPV:

(3) $$F\text{-}\text{measure}=\frac{2\times \text{sensitivity}\times \text{PPV}}{\text{sensitivity}+\text{PPV}}.$$

If there are no base-pairs in the predicted structure and the reference structure, we define PPV and Sensitivity to be 1 and otherwise 0. The F-measure, sensitivity, and PPV for the prediction of any individual structure are always in the interval [ 0,1], where 1 characterizes a perfect prediction. When assessing the prediction accuracy on a given set of structures, we usually report the average F-measure, sensitivity, and PPV achieved over the entire set.

### Statistical analysis of prediction accuracy

To formally assess the degree to which prediction accuracy results measured for a given set of RNAs depend on the precise choice of this set, we employ two well-known statistical resampling techniques: bootstrap confidence intervals and permutation tests (see, *e.g.*, [25]). Details on the respective procedures developed and used in the context of this work are described in the following. Here, we applied these statistical analysis procedures to the average F-measure determined for predictions on a given set of RNAs, but we note that they generalize in a straightforward manner to other measures of accuracy and other statistics of these over the given benchmark set. We note that these statistical techniques are limited to assessing the impact of different samples from the same underlying distribution – an important issue, considering the large variation of prediction accuracy within the sets of RNAs commonly used for evaluating structure prediction procedures – but do not allow assessment of prediction accuracy might vary as the underlying distribution is changed (*e.g.*, by modifying the relative representation of RNAs from different families or of different provenance); to address this latter question, we use a different approach described later.

To investigate the consistency of predictions obtained from different RNA secondary structure prediction procedures, we used scatter plots as well as the Spearman correlation coefficient (which, unlike the more widely used Pearson product moment correlation coefficient, also captures non-linear relationships).

#### Bootstrap percentile confidence intervals

Following common practice (see, *e.g.*, [25]), for a vector **f** of F-measure values achieved by a given prediction procedure on the structures contained in a given set *S* of RNAs (here, S-STRAND2), we perform the following steps to determine the 95% confidence interval (CI) for the mean F-measure: 

•Repeat for 10^4^ times: from **f**, draw a uniform random sample of size |**f**| with replacement, and calculate the average F-measure of the sample.

•Report the 2.5*th* and 97.5*th* percentiles of the distribution of F-measures from Step 1 as the lower and upper bounds of the CI, respectively.

The choice of 10^4^ samples in Step 1 follows standard practice for bootstrap CIs (as illustrated by the results shown in Figure S2 in the Supporting Information, the results obtained using different sample sizes are very similar).

#### Permutation test

Following common practice (see, *e.g.*, [25]), for vectors **f**_*A*_ and **f**_*B*_ of F-measure values achieved by given prediction procedures *A* and *B*, respectively, on the structures contained in a given set *S* of RNAs (here, S-STRAND2), we perform the following steps to perform a permutation test for the null hypothesis that the mean F-measure achieved by *A* and *B* is the same: 

•Repeat for 10^4^ times: For each RNA in *S*, swap the F-measures of *A* and *B* with probability 1/2, resulting in vectors $f_{A}^{\;\prime }$ and $f_{B}^{\;\prime }$, respectively.

•Construct the cumulative distribution function (CDF) of $\text{avg}(f_{A}^{\;\prime })-\text{avg}(f_{B}^{\;\prime })$ from the 10^4^ permuted pairs of vectors $f_{A}^{\;\prime }$, $f_{B}^{\;\prime }$ from Step 1, where *avg*(·) denotes the average over the elements of a given vector.

•Determine the percentile *c* of the CDF from Step 2 that is equal to *avg*(**f**_*A*_)−*avg*(**f**_*B*_), as determined from the original, unpermuted performance vectors for *A* and *B*; *p*=(100−*c*)/100 is the *p*-value of the test.

•Reject the null hypothesis of equal performance if, and only if, *p* from Step 3 is smaller than a given significance threshold *α*.

The choice of 10^4^ repetitions in Step 1 follows standard practice for permutation tests. In this work, we used this test with a standard significance threshold of *α*=0.05.

### AveRNA

As explained earlier, the key idea behind *AveRNA* is to exploit complementary strengths of a diverse set of prediction algorithms by combining their respective secondary structure predictions for a given RNA sequence. Our *AveRNA* procedure can make use of an arbritrary set of prediction procedures, **A**:={*A*_1_,*A*_2_,…,*A*_*k*_}, which it uses in a black-box manner to obtain predictions for a given input sequence, *s*. To emphasise the fact that the subsidiary structure prediction procedures in **A** are effectively just an input to *AveRNA* that can be varied freely by the user, we use AveRNA (**A**) to denote *AveRNA* with set **A** of component structure prediction procedures.

Applied to input RNA sequence *s*, AveRNA (**A**) first runs each *A*_*l*_∈**A** on *s*, resulting in predicted structures *S*(*A*_1_,*s*),*S*(*A*_2_,*s*),…,*S*(*A*_*k*_,*s*). Let each of these structures *S* be represented by a base-pairing matrix *BP*(*S*) defined by *BP*(*S*)_*i*,*j*_=1 if *i* and *j* form a base-pair in *S* and *BP*_*i*,*j*_=0 otherwise, where *i*,*j*∈{1,2,…,*n*}. We note that any RNA secondary structures, pseudo-knotted or not, corresponds to exactly one such binary matrix, but not every binary matrix represents a valid secondary structure.

We now consider the normalised sum of these binary matrices:

(4) $$P=\overset{k}{\underset{l=1}{\sum }}\frac{\text{BP}(S(A_{l},s))}{k}.$$

Each entry *B*_*i*,*j*_ of this matrix can be interpreted as the probability of a base pair between bases *i* and *j* in input sequence *s*, under the assumption that the predictions obtained from each of the *A*_*l*_ should be considered equally likely to be correct. This is equivalent to tallying votes for each possible base pair, where each predictor has one vote per candidate pair *i*,*j*.

However, it may well be that some predictors are generally more accurate than others, as is known to be the case for the set of secondary structure predictors we consider in this work. Therefore, we associate a weight (in the form of a real number between 0 and 1) with each predictor and consider the weighted normalised sum of the individual secondary structure matrices:

(5) $$P(w)=\overset{k}{\underset{l=1}{\sum }}w_{l}\cdot \text{BP}(S(A_{l},s)),$$

where **w**=(*w*_1_,*w*_2_,…,*w*_*k*_), each *w*_*l*_ is the weight assigned to predictor *l*, and $\overset{k}{\underset{i=1}{\sum }}w_{i}=1$. We note that the unweighted case from above corresponds to *w*_*l*_=1/*k* for each *l*. Before discussing the interesting question of how to determine appropriate weights, we describe in the following how we infer the pseudoknot-free RNA structure ultimately returned by *AveRNA* from the entries in the weighted probability matrix *P*(**w**).

#### Structure inference

The final structure prediction returned by AveRNA (**A**) for a given sequence can be obtained in different ways. First, we note that the problem of extracting a pseudoknot-free structure from the resulting probability matrix can be solved using a Nussinov-style dynamic programming (DP) algorithm to infer maximum expected accuracy (MEA) structures [6]. We refer to the variant of *AveRNA* that uses this procedure as **AveRN***A*_*DP*_. Unfortunately, this DP procedure requires *Θ*(*n*^3^) running time, which becomes problematic in the context of the parameter optimisation described later. Therefore, we designed the following greedy algorithm as an alternative way for estimating MEA structures. Let **p**=(*p*_1_,*p*_2_,…) be the sorted list of base-pair probabilities in *P*(**w**) in decreasing order and *V*=(*v*_1_,*v*_2_,…) be the respective set of base-pairs. For a given threshold *θ* (a parameter of the procedure whose value we discuss later), we begin with an empty set of base-pairs *S*, set *i*:=1, and repeat as long as *p*_*i*_≥*θ*: (1) Add *v*_*i*_ to *S* if (and only if) it is compatible with all other pairs in *S*, *i.e.*, does not involve a base already paired with another position or introduce a pseudoknot in *S*; (2) increment *i*. We refer to the variant of *AveRNA* using this greedy inference method as **AveRN***A*_*Greedy*_.

We note that, while the greedy inference method is not guaranteed to find a MEA structure, as we will show later, it performs very well compared to the exact DP inference algorithm and is computationally much more efficient. When either variant of *AveRNA* is applied to a set of RNA sequences, prediction and structure inference are performed for each given RNA sequence independently, and the results are independent of the composition of the set or the order in which sequences are considered.

#### Parameter optimization

Clearly, the performance of AveRNA (**A**) depends on the set **A** of component prediction procedures as well as on the previously mentioned parameters, namely the weights *w*_*l*_ and, for **AveRN***A*_*Greedy*_, the pairing threshold *θ*. Before using AveRNA (**A**) for prediction tasks, we would like to find settings for these parameters that would result in optimised prediction accuracy obtained on a set of reference RNAs (in terms of mean F-measure over the set). We solved the resulting numerical optimisation problem using a well-known procedure called *covariance matrix adaptation evolution strategy (CMA-ES)*[26,27]. CMA-ES is a non-convex, gradient-free parameter optimization procedure that has proven to be empirically successful in many real-world applications and appeared to be the most appropriate tool for finding performance-optimising parameters of *AveRNA*. We used the MATLAB implementation of CMA-ES with default settings, except that we had to increase the maximum number of iterations to 100, since in some cases we observed clear evidence that a global optimum was not reached with the lower default setting for this parameter [28].

#### Time complexity

The running time required to run AveRNA (**A**) (with a fixed set of parameters) is essentially the sum of the running times of the component prediction procedures *A*_1_,…,*A*_*k*_ (where we note that in principle, these can be run in parallel and the time required for inferring the output structure from these results). While for **AveRN***A*_*DP*_, the latter time is of order *Θ*(*n*^3^), and therefore no worse than the complexity of most RNA secondary structure prediction methods based on dynamic programming, for **AveRN***A*_*Greedy*_, it is *O*(*n*^2^) in the (unrealistic) worst case and negligible in practice.

Parameter optimisation requires substantially more computational effort, but has to be performed only once, off-line, very much like optimisation of the parameters of a given energy model. In the context of *AveRNA*_*DP*_, each iteration of this optimisation process involves running the *Θ*(*n*^3^) DP procedure on all sequences in the given training set of RNAs, and as we will demonstrate later, it turns out to be important to use reasonably large and diverse training sets. In our experiments, using a training set of 500 sequences, one iteration of CMA-ES on *AveRNA*_*DP*_ took 653 250 seconds (*i.e.,* more than 750 CPU days for the full optimization). Each iteration of optimising *AveRNA*_*Greedy*_, on the other hand, took only 2 880 seconds (*i.e.,* the full optimization required less than 4 CPU days). Note that these runtimes do not include the time required by the individual algorithms for predicting the structures, which are the same for both approaches and need to be expended only once at the beginning of the optimisation process. Once the parameters of AveRNA are optimised, it runs efficiently, as described at the beginning of this section.

### Ablation analysis

Measuring the contribution of each algorithm to AveRNAs performance can help us answer a wide range of questions, including the following: Which component prediction procedure contributes the most to the overall performance of *AveRNA*? Is there a certain number of component prediction procedures that must be included before the ensemble method outperforms the individual ones? Are there prediction procedures that can compensate for each other, in the sense that including one procedure from a certain set is important, but adding others from the same set does bring significant further gains? For AveRNA (**A**) with **A**={*A*_1_, *A*_2_,...,*A*_*k*_} we assessed the contribution of each *A*_*l*_ using the following ablation procedure: 

•Determine the *A*_*l*_∈**A** for which AveRNA (**A**∖{*A*_*l*_}) performs worst^1^, *i.e.*, whose average F-measure on the give set of RNAs is lowest.

•Remove *A*_*l*_ from Step 1 from **A**.

•If **A** still contains more than two algorithms, go to Step 1 and iterate.

Step 1 involves re-optimising the parameters of *AveRNA* for each set of component algorithms, starting from the values of AveRNA (**A**).

## Results

In our computational experiments, we pursued two major goals: firstly, to critically assess the state of the art in predicting pseudoknot-free MFE RNA secondary structures, and secondly, to demonstrate that our *AveRNA* ensemble-based structure prediction method does indeed achieve significantly better results than previous algorithms.

### Performance of existing prediction methods

**Figure 1:**
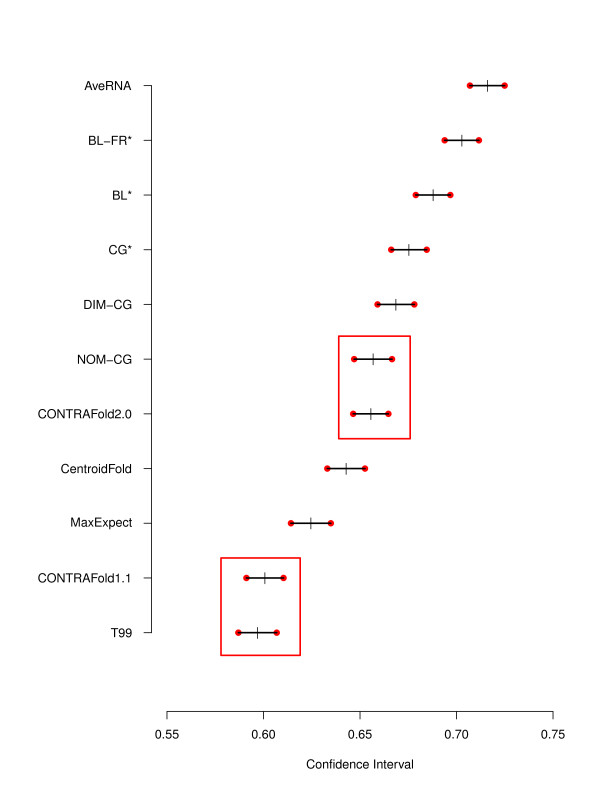
**F-measure confidence intervals.** 95% Confidence Intervals for the F-measure of different prediction algorithms (red circles) and the mean F-measure (black crosses). The red rectangles indicate algorithms with statistically insignificant performance differences, as determined by a permutation test.

Table 1 shows the the mean F-measure value for each method on the S-STRAND2 dataset, along with bootstrap confidence intervals calculated as explained in the previous section, which are also shown graphically in Figure 1. Table 2 shows the results (*p*-values) obtained from permutation tests for each pair of methods. As can be seen from this table, the only statistically insignificant performance differences were observed between *T99* and *CONTRAfold 1.1*, and between *CONTRAfold 2.0* and *NOM-CG*.

**Table 1:** Prediction accuracy for various prediction algorithms

	**Mean (CI) S-STRAND2 F-measure**	**Mean testset F-measure**	**Mean testset 2 F-measure**	**Citation**
AveRNA	0.716 (0.707, 0.725)	0.711 (0.701, 0.721)	0.725 (0.713, 0.737)	-
BL-FR*	0.703 (0.694, 0.712)	0.698 (0.687, 0.708)	0.717 (0.706, 0.729)	[5]
BL*	0.688 (0.678, 0.698)	0.686 (0.675, 0.696)	0.704 (0.692, 0.715)	[5]
CG*	0.676 (0.666, 0.685)	0.673 (0.662, 0.684)	0.690 (0.677, 0.702)	[4]
DIM-CG	0.668 (0.658, 0.678)	0.664 (0.654, 0.674)	0.681 (0.668, 0.695)	[5]
NOM-CG	0.656 (0.646, 0.667)	0.653 (0.643, 0.663)	0.667 (0.655, 0.680)	[5]
CONTRAfold2.0	0.656 (0.647, 0.665)	0.650 (0.640, 0.660)	0.657 (0.644, 0.668)	[3]
CentroidFold	0.643 (0.633, 0.652)	0.638 (0.627, 0.648)	0.643 (0.630, 0.655)	[7]
MaxExpect	0.625 (0.615, 0.635)	0.619 (0.607, 0.630)	0.633 (0.620, 0.646)	[6]
CONTRAfold1.1	0.601 (0.591, 0.610)	0.595 (0.584, 0.605)	0.605 (0.592, 0.619)	[3]
T99	0.597 (0.587, 0.607)	0.591 (0.581, 0.602)	0.606 (0.593, 0.619)	[1]

F measures and 95% confidence intervals, calculated using bootstrapping, and shown in parentheses.

**Table 2:** Spearman correlation for pairs of prediction algorithms

	**AveRNA**	**BL-FR**^**∗**^	**BL**^**∗**^	**CG**^**∗**^	**DIM-CG**	**NOM-CG**	**CONTRAfold2.0**	**CentroidFold**	**MaxExpect**	**CONTRAfold1.1**	**T99**
AveRNA											
BL-FR*	0.942										
BL*	0.886	0.857									
CG*	0.814	0.774	0.821								
DIM-CG	0.828	0.764	0.819	0.897							
NOM-CG	0.788	0.747	0.801	0.899	0.877						
CONTRAfold2.0	0.769	0.707	0.716	0.733	0.749	0.722					
CentroidFold	0.758	0.698	0.714	0.715	0.741	0.715	0.937				
MaxExpect	0.749	0.689	0.730	0.732	0.769	0.751	0.755	0.759			
CONTRAfold1.1	0.720	0.660	0.685	0.707	0.733	0.719	0.799	0.818	0.780		
T99	0.703	0.665	0.691	0.687	0.697	0.728	0.670	0.684	0.749	0.691	

Spearman correlation coefficients for the F-measure values of of pairs of prediction algorithms over the S-STRAND2 dataset.

Consistent with previous work [5], we found that the oldest algorithm, *T99*, achieves a mean F-measure just below 0.6. *CONTRAfold 1.1* performs slightly better than T99 on our benchmark set, but the performance advantage is not statistically significant; we believe that the reason for this lies primarily in the fact that it was trained on a small set of RNAs not representative of the broad range of structures found in S-STRAND2. *MaxExpect* and *Centroidfold* do perform significantly better than *T99*, but fall short of the performance achieved by *CONTRAfold 2.0*. The latter method was trained on the S-STRAND2 dataset, which partly explains why it, exactly like *NOM-CG*, achieves an average F-measure that is 0.026 higher than that of *CONTRAfold 1.1*.

The methods recently developed by Andronescu *et al.*, *DIM-CG*, *CG*^∗^, *BL*^∗^ and *BL-FR*^∗^, each achieve significantly better performance than any of the previously mentioned methods; although the confidence intervals obtained for these methods show some overlap, the respective differences in mean F-measure are all significant. The best of these methods, *BL-FR*^∗^, represents an improvement of more than 0.1 in average F-measure over T99, and of almost 0.05 over *CONTRAfold 2.0*.

### Performance correlation

**Figure 2:**
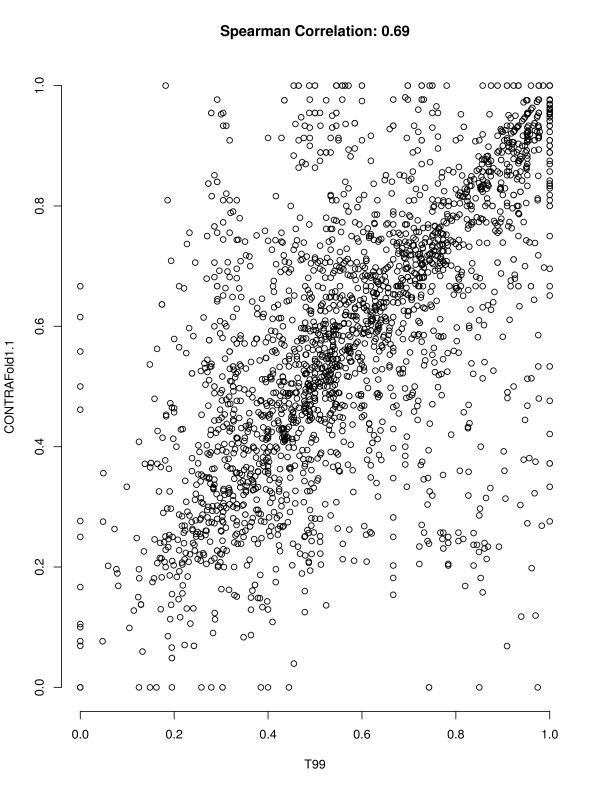
**Scatter plot of F-measures of *T99 *and *CONTRAfold 1.1.*** Correlation between the F-measure achieved by *T99* and *CONTRAfold 1.1* on the RNAs from the S-STRAND2 dataset. The mean F-measures of these algorithms are not significantly different, but prediction accuracy on individual RNAs is only weakly correlated.

**Figure 3:**
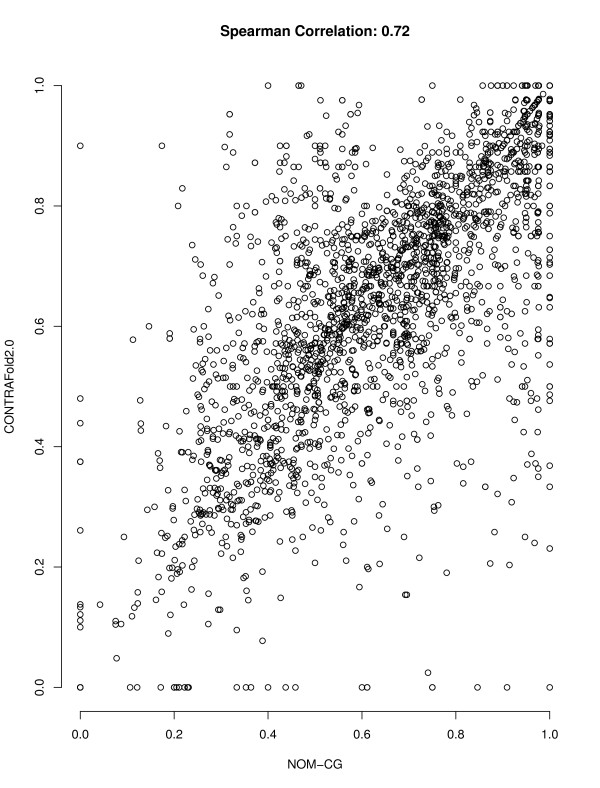
**Scatter plot of F-measures of *NOM-CG *and *CONTRAfold 2.0.*** Correlation between the F-measure achieved by *NOM-CG and CONTRAfold 2.0* on the RNAs from the S-STRAND2 dataset. The mean F-measures of these algorithms are not significantly different, but prediction accuracy on individual RNAs is only weakly correlated.

For an ensemble-based approach like *AveRNA* to work well, the set of component prediction algorithms need to have complementary strengths, as reflected in less-than perfect correlation of prediction accuracy over sets of RNA sequences. As can be seen in Table 2, the pairwise performance correlation between the procedures we considered in our study is not very strong (as indicated by Spearmann corelation coefficients between 0.66 and 0.86). Figures 2 and 3 illustrate this further by showing the correlation in F-measure across our set of RNAs for the two pairs of algorithms whose average performance does not differ significantly, *T99* and *CONTRAfold 1.1*, and *CONTRAfold 2.0* and *NOM-CG*, respectively. (In these scatter plots, each data point corresponds to one RNA from our S-STRAND2 set.)

### Performance of AveRNA

After optimizing the weights on our training set of RNAs, we found that there was no statistically significant difference between the predictions of *AveRNA*_*DP*_ and *AveRNA*_*Greedy*_ on the S-STRAND2 set (as determined using a permutation test, which yielded a *p*-value of 0.51). Because of its substantially lower run-time requirements, especially during training, we therefore decided to focus on *AveRNA*_*Greedy*_ for the remainder of our study, and we refer to this variant simply as *AveRNA*.

As can be seen in Table 1, *AveRNA* achieved an average F-measure of 0.716 on S-STRAND2, compared to 0.703 obtained by the best previous method, *BL-FR*^∗^.

Moreover, even when assessing *AveRNA* on a test set obtained by excluding the 500 sequences used for parameter optimisation from S-STRAND2, it achieves significantly higher prediction accuracy than any of its constituent algorithms. We note that although this performance improvement might appear to be modest, it is about as much as the difference between *BL*^∗^ and *BL-FR*^∗^ and, according to a permutation test, statistically highly significant (see Table 3).

**Table 3:** Pairwise permutation tests between prediction algorithms

	**AveRNA**	**BL-FR**^**∗**^	**BL**^**∗**^	**CG**^**∗**^	**DIM-CG**	**NOM-CG**	**CONTRAfold2.0**	**CentroidFold**	**MaxExpect**	**CONTRAfold1.1**	**T99**
AveRNA											
BL-FR*	0										
BL*	0	0									
CG*	0	0	0.0001								
DIM-CG	0	0	0	0.0002							
NOM-CG	0	0	0	0	0						
CONTRAfold2.0	0	0	0	0	0.0001	**0.4193**					
CentroidFold	0	0	0	0	0	0.0001	0				
MaxExpect	0	0	0	0	0	0	0	0			
CONTRAfold1.1	0	0	0	0	0	0	0	0	0		
T99	0	0	0	0	0	0	0	0	0	**0.1317**	

P-values obtained from permutation tests to determine the statistical significance of performance differences (in terms of F-measure over the S-STRAND2 dataset) between prediction algorithms. All p-values larger than a standard significance threshold of 0.05 are bolded, indicating cases where the performance differences are insignificant.

To study *AveRNA*’s performance on sets of RNAs of different types and provenance, we optimised the parameters for *AveRNA* on subsets of S-STRAND2, from which one of the 7 classes that make up the RNA STRAND database had been excluded, and then tested on the excluded class only, such that there was not only no overlap between training and test set, but also very little similarity. This is a situation where many machine learning techniques are known to perform quite poorly. The results from this experiment, shown in Table 4, indicate clearly that, even in this very challenging setting, *AveRNA* performs very well: only on 2 of the 7 classes, *AveRNA* performs significantly worse if trained under exclusion of that class, and in the two remaining cases, the loss in accuracy was only about 2% (Additional file 1: Table S1 for detailed results from the respective permutation tests).

**Table 4:** Class-specific prediction accuracy for various prediction algorithms

	**ALL**	**ASE**	**CRW**	**PDB**	**RFA**	**SPR**	**SRP**	**TMR**
n	2511	386	411	311	257	526	350	269
Testset contribution	0.8	0.83	0.79	0.76	0.78	0.78	0.80	0.87
Mean sequence length	332	959	75	129	116	77	226	362
BL-FR*	0.703	0.606 (0.592, 0.620)	0.613 (0.590, 0.637)	0.900 (0.878, 0.920)	0.674 (0.633, 0.713)	0.780 (0.761, 0.800)	0.734 (0.712, 0.755)	0.589 (0.569, 0.607)
BL*	0.688	0.604 (0.589, 0.618)	0.583 (0.561, 0.603)	0.894 (0.871, 0.915)	0.667 (0.627, 0.704)	0.763 (0.742, 0.782)	0.717 (0.693, 0.738)	0.568 (0.550, 0.587)
CG*	0.676	0.601 (0.588, 0.615)	0.576 (0.556, 0.597)	0.891 (0.868, 0.911)	0.640 (0.604, 0.675)	0.791 (0.771, 0.809)	0.675 (0.651, 0.698)	0.496 (0.477, 0.515)
DIM-CG	0.668	0.605 (0.592, 0.618)	0.559 (0.540, 0.577)	0.885 (0.863, 0.906)	0.661 (0.625, 0.696)	0.785 (0.765, 0.804)	0.655 (0.630, 0.680)	0.470 (0.451, 0.488)
NOM-CG	0.656	0.602 (0.588, 0.616)	0.568 (0.547, 0.587)	0.885 (0.862, 0.905)	0.637 (0.603, 0.674)	0.739 (0.719, 0.760)	0.660 (0.635, 0.685)	0.457 (0.438, 0.476)
CONTRAfold2.0	0.656	0.651 (0.639, 0.664)	0.550 (0.532, 0.568)	0.869 (0.846, 0.891)	0.607 (0.569, 0.645)	0.746 (0.729, 0.763)	0.609 (0.587, 0.633)	0.509 (0.488, 0.527)
CentroidFold	0.643	0.642 (0.630, 0.654)	0.537 (0.517, 0.556)	0.860 (0.833, 0.885)	0.607 (0.568, 0.646)	0.705 (0.683, 0.724)	0.623 (0.600, 0.646)	0.492 (0.473, 0.512)
MaxExpect	0.625	0.577 (0.564, 0.589)	0.508 (0.488, 0.527)	0.858 (0.828, 0.883)	0.644 (0.611, 0.680)	0.695 (0.673, 0.715)	0.634 (0.608, 0.659)	0.435 (0.417, 0.452)
CONTRAfold1.1	0.601	0.590 (0.578, 0.602)	0.440 (0.421, 0.459)	0.841 (0.817, 0.866)	0.597 (0.565, 0.630)	0.690 (0.669, 0.712)	0.619 (0.594, 0.643)	0.392 (0.374, 0.410)
T99	0.597	0.546 (0.531, 0.560)	0.502 (0.481, 0.522)	0.860 (0.833, 0.885)	0.625 (0.594, 0.657)	0.583 (0.563, 0.604)	0.689 (0.666, 0.710)	0.389 (0.371, 0.406)
AveRNA	0.716	0.653 (0.641, 0.665)	0.618 (0.600, 0.638)	0.906 (0.884, 0.925)	0.683 (0.645, 0.719)	0.794 (0.776, 0.812)	0.732 (0.707, 0.753)	0.592 (0.575, 0.608)
AveRNA-I		0.676 (0.663, 0.687)	0.619 (0.602, 0.639)	0.901 (0.878, 0.922)	0.673 (0.640, 0.707)	0.808 (0.789, 0.825)	0.736 (0.715, 0.757)	0.590 (0.569, 0.608)
AveRNA-E		0.650 (0.637, 0.663)	0.617 (0.597, 0.637)	0.907 (0.885, 0.926)	0.683 (0.646, 0.718)	0.794 (0.774, 0.811)	0.710 (0.688, 0.733)	0.573 (0.555, 0.589)

F-measure values for different algorithms for different classes in the S-STRAND2 dataset. AveRNA-I has been trained on 20% of the given class sampled uniformly at random, and the overall F-measure for the entire class is reported. AveRNA-E has been trained on 20% of S-STRAND2 excluding the given class, and the F-measure for the given class is reported.

We further note that, as per the results shown in Table 4, prior to *AveRNA*, the best energy-based prediction algorithm varied between RNA classes. On the other hand, *AveRNA* was found to not perform significantly worse than the previous best method on any of the 7 classes, and in 2 of them (CRW and RFA - see Additional file 1: Table S1), it performed significantly better. This suggests (but of course cannot guarantee) that *AveRNA* is likely to perform at least as well as other general purpose energy-based secondary structure prediction algorithms on previously unseen classes of RNAs. Furthermore, we also optimised *AveRNA* on a small part of each of the 7 classes and then evaluated it on the entire class; the results of this experiment, also shown in Table 4, indicate that by training a generic version on the broader set of sequences described earlier gives surprisingly good and robust performance – only for 3 of the 7 classes (ASE, SPR, and SRP) the respective class-specific version of *AveRNA* performs significantly better and in one class (PDB) it performs worst. Table 4 also shows the mean sequence length for every class of RNAs and provides clear evidence that *AveRNA*’s performance relative to its constituent algorithms does not deteriorate with increasing sequence length.

One interesting property of *AveRNA* (**A**) is that the trade-off between sensitivity and PPV can be easily and intuitively controlled by the threshold *θ*∈ [ 0,1]: For high *θ*, only base pairs are predicted for which there is high agreement between the procedures in **A**, and therefore, we expect relatively few false positive predictions at the cost of relatively many false negatives, while for low *θ*, even base pairs predicted by very few procedures in **A** tend to be included in the overall prediction, leading to relatively many false positive, but few false negatives. *CONTRAfold 1.1*, *CONTRAfold 2.0*, *Centroidfold* and *MaxExpect* also afford control of this trade-off, via the parameter *γ*∈ [−5,6], but in a less intuitive manner.

**Figure 4:**
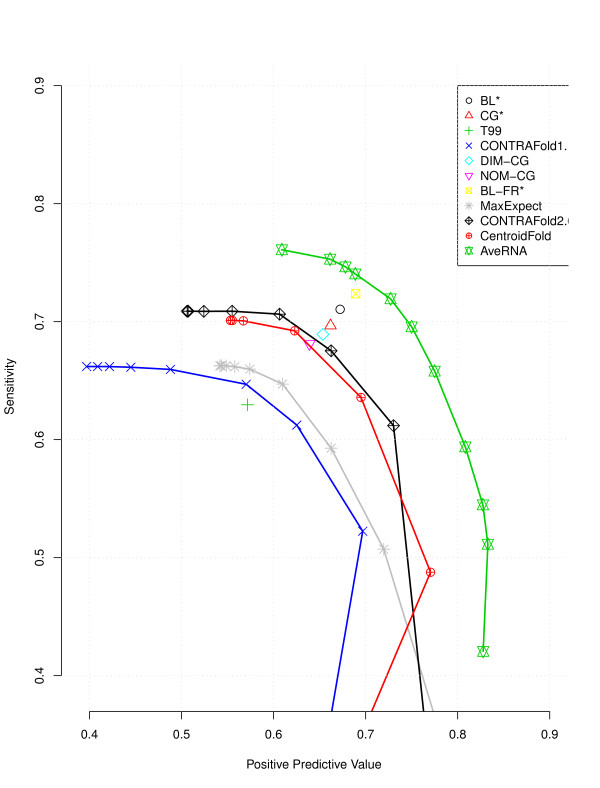
**Sensitivity versus PPV.** Sensitivity *vs* positive predictive value (PPV) for different prediction algorithms; for *AveRNA*, the points along the curve were obtained by adjusting the pairing threshold *θ*, and for *CONTRAfold 1.1, CONTRAfold 2.0, Centroidfold and MaxExpect* by adjusting the parameter *γ*.

Figure 4 illustrates the trade-off between sensitivity and PPV for all of these algorithms and shows clearly that overall, *AveRNA* dominates all previous methods, and in particular, gives much better results than the previous best algorithm that afforded control over this trade-off, *CONTRAfold 2.0*. We note that, in all cases, as a procedure becomes increasingly more conservative in predicting base pairs, eventually, both sensitivity and PPV drop (see Additional file 1: Figure S1); we believe this to be a result of the high detrimental impact of even a small number of mispredicted base pairs when overall very few pairs are predicted.

### Ablation analysis

The results of the ablation analysis we conducted to study the relative impact of the various component prediction procedures in **A** on the performance of *AveRNA* (**A**) are shown in Table 5. The top 11 rows contain the weights assigned to each algorithm; cases in which a procedure from **A** was dropped during the optimisation process are indicated by a value of zero. The bottom three rows show the value of threshold *θ* and the average performance on the training and test sets, respectively.

**Table 5:** Ablation analysis results

	**0**	**1**	**2**	**3**	**4**	**5**	**6**	**7**	**8**	**9**
BL-FR*	40.8030									
BL*	3.4339	36.1240								
CG*	0.5814	28.3500	23.6200							
DIM-CG	13.3610	2.2809	18.8470	25.2980						
NOM-CG	0	1.4514	7.5372	19.4300	29.6720					
CONTRAfold2.0	7.9964	20.2750	24.4660	25.1060	34.6240	48.6310				
CentroidFold	6.7425	0.0103	16.4620	15.9370	4.8337	11.1500	48.8500			
MaxExpect	18.0520	0	3.8522	14.2290	18.5270	24.7580	5.6026	24.0080		
CONTRAfold1.1	1.8412	8.4554	0	0	3.3164	5.2330	16.9320	42.9650	62.8050	
T99	7.1883	3.0532	5.2156	0	9.0275	10.2280	28.6160	33.0280	37.1950	100
Threshold	42.7290	38.8610	35.6670	36.8770	31.2980	34.4810	31.6520	50	50	50
F (train)	0.7350	0.7163	0.7106	0.7052	0.7002	0.6889	0.6798	0.6640	0.6271	0.6188
F (test)	0.7158	0.7050	0.6948	0.6886	0.6842	0.6718	0.6629	0.6423	0.6011	0.5967

Each data column corresponds to one stage of the ablation analysis, with the (optimised) weights of each prediction algorithm included in the ensemble shown in the top part of the table, followed by the (optimised) pairing threshold and the training and testing performance (in terms of mean F-measure) in the bottom part.

It is interesting to note that although *BL-FR*^∗^ has a weight of over 40% in the full ensemble, excluding it leads to a rather modest drop of only 0.011 in average F-measure, and this drop in performance is the highest caused by removing any single procedure from the full set **A**. Similarly, the decreases in performance as additional procedure are removed, are mostly quite small. This indicates that, within the set of prediction procedures we considered here, there is not only sufficient complementarity in the strength of individual procedures to obtain benefits from the ensemble-based approach, but also enough similarity in strength between some of the procedures to permit compensating for the removal of one by increasing the weight of others.

As seen in Table 5, up to the point where only one procedure is left in **A**, the performance of *AveRNA* (**A**) is always higher than that of any of its constituents, indicating the efficacy and robustness of our ensemble-based prediction approach.

### Training set selection

Clearly, *AveRNA*’s performance depends on the training set that is used as a basis for optimising its weight parameters. To study the effect of training set size on performance (in terms of mean F-measure), we generated 11 training sets of size 100 and 200, as well as one training set of size 500 and one set of size 1000. We then optimised *AveRNA* (**A**) for each of these sets and measured the performance obtained on the full S-STRAND2 test set. As can be seen from the results of this experiment shown in the Table 6, decreasing the training set size from 500 to 200 lead to a modest drop in mean F-measure by 0.004, and further decrease to 100 caused a larger drop by 0.007. On the other hand, increasing the size of the training set from 500 to 1000 merely resulted in a very small performance improvement of less than 0.001. This indicates that, while it is important to use a reasonably large and diverse training set, at least for the set of prediction procedures considered here, there is only very limited value in using sets larger than that of size 500 we used for all other experiments.

**Table 6:** Impact of training set size on prediction accuracy

**Training set size**	**F-measure**	**CI**
1000	0.7175	(0.7095, 0.7278)
500	0.7167	(0.7075, 0.7269)
200	0.7131 (0.7108, 0.7140)	(0.7041, 0.7236)
100	0.7061 (0.7050, 0.7127)	(0.6943, 0.7184)

Mean F-measure on S-STRAND2, with 95% confidence intervals shown in the last column. For the bottom two rows, training set size was sampled 11 times uniformly at random, and the median (20-, 80-percentiles) of the prediction accuracies from these samples are reported, along with confidence intervals for the medians.

We note that we did not use the training set developed by Andronuescu *et al.* (2010) in the context of energy parameter estimation, primarily because many of the prediction procedures we study here have been optimised on that set (which could have biased *AveRNA* to assign higher weights to those algorithms and lead to poor generalization to test data). We also note that all training sets we considered were obtained by random uniform sampling from the full S-STRAND2 set.

Additionally, in Table 2 we have reported the F-measures of testset2, a new testset which consists of all members of S-STRAND2 which have not been used by *AveRNA* or any of the individual algorithms for training purposes. Permutation tests on this new test set (Table S2) confirm that *AveRNA* remains significantly more accurate than the other algorithms.

## Discussion

To no small extent, our work presented here was motivated by the observation that in many cases, the differences in accuracy achieved by RNA secondary structure prediction methods are quite small on average, but tend to vary very significantly between individual RNAs [5,6]. While this is not surprising, it suggests that care should be taken when assessing different prediction methods to ensure statistically meaningful results, and that potentially, benefits could be derived from combining predictions obtained from different methods. The statistical procedures we use in this work make it possible to assess statistical significance in a well-established, quantitative and yet computationally affordable way, and our *AveRNA* procedure provides a practical way for realising the benefits inherent in a set of complementary prediction methods.

Our results demonstrate that there has, indeed, been steady progress in the prediction accuracy obtained from energy-based RNA secondary structure prediction methods. The fact that *CONTRAfold 1.1* provides no statistically significant improvement in accuracy over the standard *T99* energy model when both are evaluated on our large and diverse set of reference structures needs to be viewed in light of the fact that *CONTRAfold 1.1* was trained on a limited set of RNA structures from the RFam database. The fact that *CONTRAfold 2.0*, which was trained on the the same larger and richer set used by Andronescu *et al.*[4], performs much better further highlights the importance of the training set used as a basis for empirically optimising the performance of prediction methods. It is interesting to observe that the performance difference between *CONTRAfold 2.0* and *NOM-CG*, which are trained on the same set of references structures, are insignificant, which indicates that both methods are equally effective in making use of the information inherent in this set. However, *NOM-CG*, thanks to its additional use of thermodynamic data, produces a physically plausible energy model, while the probabilistic model underlying *CONTRAfold 2.0* does not produce realistic free energy values.

We further interpret the fact that *DIM-CG*, *CG*^∗^, *BL *^∗^ and *BL-FR*^∗^ all perform significantly better than *CONTRAfold 2.0* as evidence that the thermodynamic data used by the former methods can effectively inform methods for optimising prediction accuracy based on data. Our statistical analysis provides further support for the claim that the computationally more expensive Boltzmann Likelihood parameter estimation method leads to better results than the Constraint Generation method, and that the additional use of probabilistic feature relationships enables further significant improvements [5].

The accuracy results we obtained for the *MaxExpect* procedure [6] and for *Centroidfold*[7] are markedly lower than those reported in the respective original studies, mainly because our evaluation is based on a more extensive set of reference structures. However, we note that the underlying approaches of maximizing expected base-pair accuracy and *γ*−centroid estimators can in principle be applied to any prediction method that produces probability distributions over the secondary structures of a given sequence. We therefore expect that these ideas can eventually be used in combination with parameter estimation methods, such as the ones that gave rise to the *CG *^∗^, *BL*^∗^ and *BL-FR*^∗^ parameter sets.

The results of our correlation analysis revealed that prediction methods whose accuracy over the entire benchmark set does not differ much (such as *T99* and *CONTRAfold* 1.1) show large differences in accuracy on many individual RNAs. Consistent with earlier observations that predictions that are slightly suboptimal according to a given energy model can sometimes be much more accurate (see, *e.g.*, [6]), we conjecture that this is a consequence of systematic weaknesses (such as the lack of accounting for interactions between non-neighbouring bases or the use of an overly simplistic energy model for multiloops) and inaccuracies (for example, in thermodynamic measurements) in the energy models underlying these procedures. Particularly when using automated methods for optimising the parmaters of a given energy models, such weaknesses and inaccuracies could easily lead to multiple solutions that show similar performance on average, but give very different results on many individual RNAs.

This situation, while at the first glance somewhat unsatisfactory, provides the basis for our *AveRNA* approach, which obtains more accurate predictions by means of weighted combination of the predictions obtained from a set of given prediction procedures. While our study is focussed on the prediction of pseudoknot-free MFE structures, we note that the weighted sum calculation performed by *AveRNA* on base pairing matrices naturally extends to methods that produce base pairing probabilities and to pseudoknotted prediction methods. In the latter case, the calculation of the weighted probability matrix *P*(**w**) proceeds exactly as in the pseudoknot-free case, but the procedure used for structure inference would have to be modified to produce pseudoknotted MEA structures. In the former case, probability matrices are used instead of Boolean matrices, and the result of the calculation would be normalised to yield a well-formed base pairing probability matrix. (We note that, in light of very recent empirical results based on the statistical approach first developed in the context of the work presented here, it is not clear that MEA structures determined from individual base pairing probability matrices are generally more accurate than MFE structures for the same energy model [29]; however, it is possible that higher accuracies can be obtained via ensemble-based MEA predictions from weighted combinations of multiple base pairing matrices.) We pursued neither of these directions here, because currently, the number of high-accuracy prediction procedures for pseudoknotted RNA structures of base-pair probabilities is more limited and because the development and assessment of extensions of *AveRNA* to those cases pose challenges that are beyond the scope of this work, but we strongly believe that these directions are very promising and should be explored further in the future.

We note, however, that *AveRNA* as presented here already realises an advantage usually found only in approaches that produce base pairing probabilities: an easy and intuitive way for assessing the confidence with which certain bases are predicted to pair or remain unpaired, by means of inspecting the entries of the probability matrix *P*(**w**). Values close to one indicate base pairs that are predicted consistently by many of the underlying prediction procedures, and values close to zero indicate bases that are consistently predicted to be unpaired. Intermediate values indicate base pairings for which there is more disagreement between the given prediction procedures. From the fact that by thresholding these values, the sensitivity and specificity (PPV) for predicting base pairs can be increased quite substantially (as seen in Figure 3), we conclude that the set of prediction procedure used by *AveRNA* in this work is sufficiently diverse to allow for this interpretation. The threshold parameter *θ* controls the trade-off between sensitivity and PPV in an intuitive way. It is conceivable that even higher sensitivity and PPV values can be obtained by optimising the weight parameters of *AveRNA* specifically for that purpose (something we did not attempt in this work).

## Conclusions

The ensemble-based RNA secondary structure prediction method AveRNA introduced in this work not only improves over existings state-of-the-art energy-based methods, but also holds much promise for the future. *AveRNA* can make use of arbitrary secondary structure prediction procedures; in particular, as demonstrated here, it can be used to combine both MEA and MFE structures. We expect that by adding new prediction procedures to the set used by *AveRNA*, even better ensemble-based predictions can be obtained. It is conceivable that eventually, a prediction procedure becomes available that dominates all previous methods, in the sense that it provides predictions as least as accurate as these on all RNAs of interest, and in that case, the ensemble-based prediction approach of *AveRNA* would not realise any additional gains. Based on our assessment of existing methods, and considering the weaknesses and inaccuracies known to exist in all current energy models, we do not expect this situation to arise in the foreseeable future. The results of our ablation analysis further supports the view that further increases in prediction accuracy achieved by the ensemble-based prediction approach underlying *AveRNA* are likely to arise as new prediction procedures become available, since – as seen in Table 5 – that was the case when adding new procedures to sets of previously known procedures in the past.

In fact, *BL-FR*^∗^ was introduced when *AveRNA* was under development and achieved an F-measure of higher than the version of *AveRNA* available at that time. Including *BL-FR*^∗^ in *AveRNA* produced the version of *AveRNA* studied here, which – as expected – performs significantly better than *BL-FR*^∗^. This suggests that *AveRNA* not only represents the state of the art in secondary structure prediction at the time of this writing, but is likely to remain so, as improved prediction algorithms and energy models are developed and added to the generic ensemble-based approach.

It should be noted, however, that in cases where additional information about the specific secondary structure of a particular RNA is available (*e.g.*, in the form of SHAPE or other footprinting data), prediction methods that utilise this information should be expected to achieve higher accuracies (see, *e.g.*, [30]).

We see several avenues for future work: Here, we focused on pseudoknot free structures, but the general framework (except the dynamic programming) can be applied to pseudoknotted structures as well once a wider range of these algorithms are developed. Similarly, our framework can be applied to algorithms that are able to calculate base-pair probabilities (*e.g.,* based on partition functions) or to algorithms that are able to predict several sub-optimal structures. New algorithms (*e.g.,* non-energy-based methods) or different configurations of the existing algorithms (using different training strategies) can be included in *AveRNA*. We showed that the correlation between the predictions of different algorithms is not very strong. These algorithms can be studied to identify their strengths and weaknesses to provide guidance to the end-users. Alternatively, this information could be used to design an *instance-based selection algorithm* that instead of combining the predictions of all of the algorithms, either selects the most suitable algorithm for each sequence or selects a number of candidates for *AveRNA* to combine.

## Competing interests

Both authors declare that they have no competing interests.

## Authors’ contributions

HH conceived the original idea. NA and HH designed the methodology, conceived the experiments, interpreted the results, and wrote the manuscript. NA implemented the methodology and performed the experiments. All authors read and approved the final manuscript.

## Supplementary Material

Additional file 1 **Supplemental Information.** A PDF file with supplementary figures and tables as described in the main text. Click here for file
