# Supplementary material for: Ensemble-based prediction of RNA secondary structures
Source: BMC Bioinformatics. 2013 Apr 24;14:139. doi: 10.1186/1471-2105-14-139 (PMC3750279; doi:10.1186/1471-2105-14-139)
Supplement: Additional file 1 — Supplemental Information. A PDF file with supplementary figures and tables as described in the main text. [file 1471-2105-14-139-S1.pdf]

Ensemble-based Prediction of RNA Secondary Structures: Supporting Information

Nima Aghaeepour and Holger H. Hoos

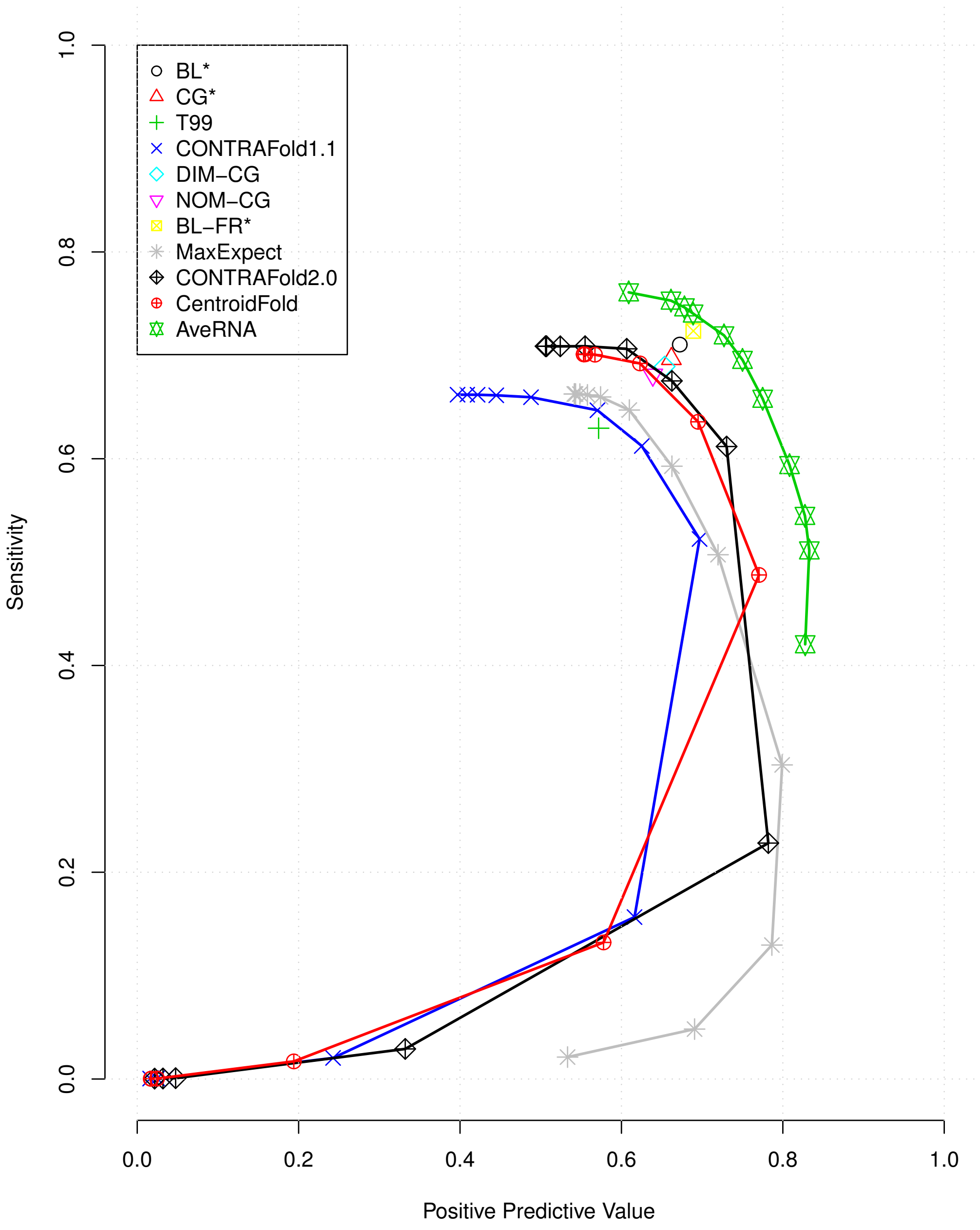

Figure S1: Same data as shown in Figure 2 of the manuscript, but with wider sensitivity and PPV ranges.

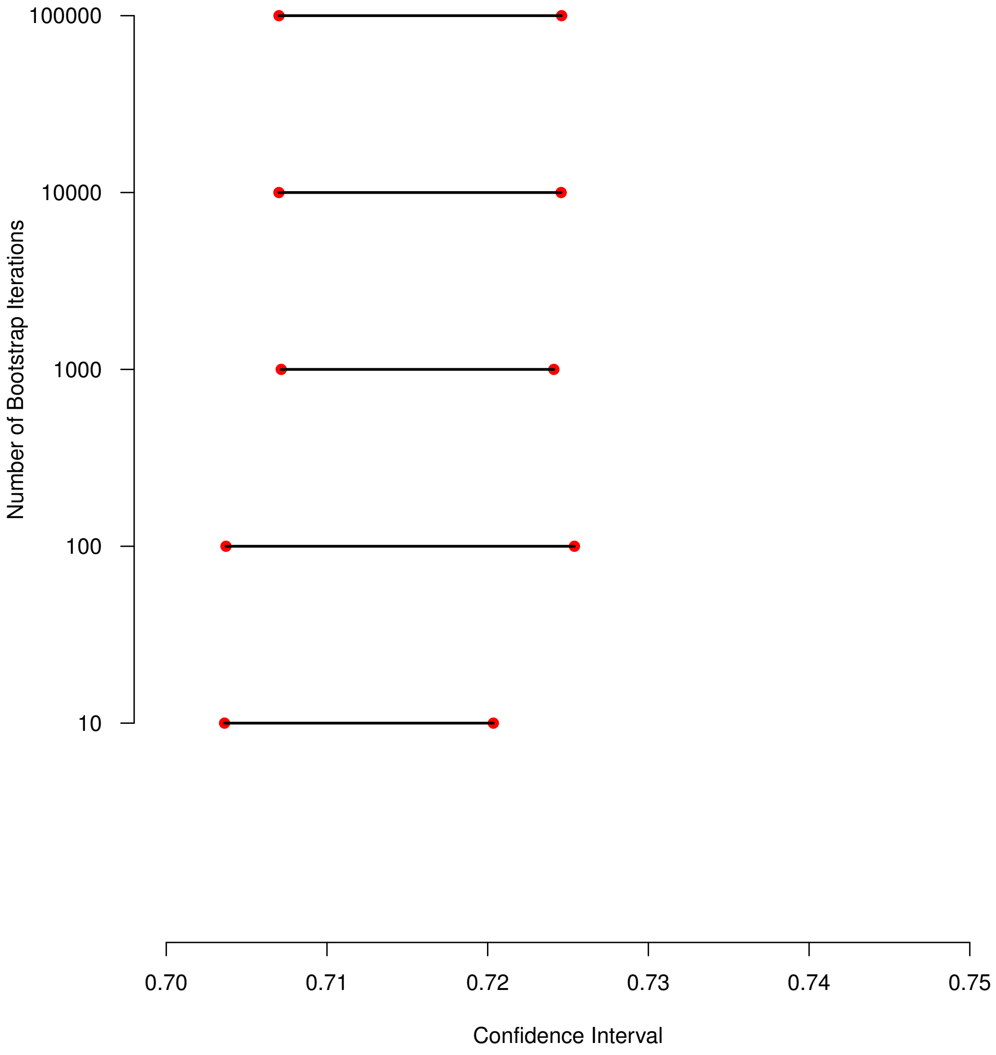

Figure S2: The confidence intervals for AveRNA's mean F-measure on S-STRAND2 for different numbers of bootstrap samples.

	ASE	CRW	PDB	RFA	SPR	SRP	TMR
AveRNA-I	0	0.4155	0.0390	0.0815	0.0255	0.0145	0.2679
AveRNA-E	0.0597	0.1937	0.3987	0.4395	0.4689	0	0
Best in Class	0.3428	0.0550	0.0112	0.0102	0.3451	0.3382	0.2598

Table S1: Permutation tests between AveRNA and a given algorithm on different components of S-STRAND2. For every class, two tests were performed to determine if AveRNA's mean F-measure is larger and smaller than that of the given algorithm. The minimum of the two obtained p-values are reported. The best in class algorithm is the one with maximum F-measure for that class in S-STRAND2 excluding AveRNA.

	AveRNA	BL-FR*	BL*	CG*	DIM-CG	NOM-CG	CONTRAFold2.0	CentroidFold	MaxExpect	CONTRAFold1.1	T99
AveRNA											
BL-FR*	0.0000										
BL*	0.0000	0.0000									
CG*	0.0000	0.0000	0.0001								
DIM-CG	0.0000	0.0000	0.0000	0.0011							
NOM-CG	0.0000	0.0000	0.0000	0.0000	0.0000						
CONTRAFold2.0	0.0000	0.0000	0.0000	0.0000	0.0000	0.0110					
CentroidFold	0.0000	0.0000	0.0000	0.0000	0.0000	0.0000	0.0000				
MaxExpect	0.0000	0.0000	0.0000	0.0000	0.0000	0.0000	0.0000	0.0000			
CONTRAFold1.1	0.0000	0.0000	0.0000	0.0000	0.0000	0.0000	0.0000	0.0000	0.0000		
T99	0.0000	0.0000	0.0000	0.0000	0.0000	0.0000	0.0000	0.0000	0.0000	0.0000	

Table S2: P-values for permutation tests over every pair of algorithms in Test Set 2.
